# Decentralised clinical training in optometry: a developmental strategy for eye health in KwaZulu Natal?

**DOI:** 10.1017/S1463423618000397

**Published:** 2018-06-20

**Authors:** Diane van Staden

**Affiliations:** Discipline of Optometry, University of KwaZulu Natal, Durban, South Africa

**Keywords:** community-based training, decentralised clinical training, human resources for eye health, optometry, primary health care

## Abstract

**Background:**

Decentralised clinical training (DCT) in optometry is an emerging concept in South Africa. In 2016, the University of KwaZulu Natal (UKZN) implemented this adaptive model of clinical training for undergraduate health professions. The initiative, which emanated through an agreement between UKZN’s College of Health Sciences and the KwaZulu Natal Department of Health, centres on the placement of undergraduate optometry students within public health facilities for clinical training purposes. Optometry services in South Africa have, however, had a historical bias towards a private sector model of training and a curative practice approach resulting in access barriers for the rural poor and high levels of unmet need. It has further contributed to a general state of underdevelopment of eye health services within the public sector.

**Discussion:**

DCT challenges historical undergraduate programme structures and modes of teaching and learning in optometry. It is largely underpinned by a need to strengthen health service delivery through a primary health care-centred, community-based training approach and produce ‘fit-for-purpose’ graduates who have contextually appropriate competencies for effective, local health service delivery. The historical absence of optometry services within the public sector has, however, contributed to limited planning for, and development of eye health services in this sector. This has inadvertently contributed to the burden of avoidable vision impairment in the country. The public health system in South Africa, therefore, faces various developmental challenges which impact eye health services and student clinical training.

**Conclusion:**

While the model is still in a developmental state and resourcing challenges potentially affecting DCT are noted, early experiences of the Discipline of Optometry at the UKZN are that DCT shows promise in terms of its potential contribution towards the development of eye health services within the public health sector from graduate readiness, resource strengthening, access improvement and health service development perspectives.

## Background

The profession of optometry in South Africa has historically been private sector-biased in both its training and practice approaches. Coupled with the country’s long history of an inequitable, fragmented health care system, maldistribution of resources and services continues to challenge eye health service delivery nationally (Moorman *et al*., 2014) despite the public health system being transformed into an integrated, national health service (Coovadia *et al*., 2009). More recently, the National Department of Health announced a re-engineering of primary health care (PHC) as a means of improving access and strengthening health services towards improved health outcomes (Barron, 2011). However, the integration of eye health into PHC is generally weak across the African continent (Du Toit *et al*., 2013). Specialised health services such as optometry remain concentrated in more developed areas, with sporadic outreach services to some rural communities (McIntyre *et al*., 2008), resulting in a huge unmet need.

Africa has long suffered a disproportionate burden of blindness and vision impairment, linked with issues of poverty and underdevelopment (Dandona and Dandona, 2001). Local census data confirms that the burden of vision impairment is greatest in more rural areas in South Africa (Statistics South Africa, 2011) where access to eye health services is limited (Mashige *et al*., 2015). Furthermore, vision impairment has health and economic consequences (Fricke, 2012). Conversely, therefore, by addressing eye health service needs, efforts by the government to improve the overall development status of communities and the country as a whole will be supported. However, with the majority of optometrists in South Africa still practicing within the private sector (Maake, 2014), human resources for eye health within public health facilities remains constrained.

The higher education sector in South Africa also faces challenges in its attempts at redress (Badat, 2010), with increased access opportunities for previously disadvantaged groups placing pressure on capacity limits at higher education institutions. Additionally, there is growing engagement within academic circles around the applicability of historical curricula and models of training given the changing dynamic in student profile, as well as current national context and needs. Therefore, transformation within the sector aligned to democratic South Africa’s evolving reality is imperative. The training of health professionals in particular has come under the spotlight in the recent past, in line with the global movement around transforming health professions education as an imperative to strengthening health systems (Frenk, 2010). Initiatives such as the SUCCEED (2015) project have been working with universities to develop models for decentralised undergraduate medical training, aimed at transforming medical education and strengthening human resources for health in South Africa.

In 2014 the University of KwaZulu Natal (UKZN), in collaboration with the KwaZulu Natal Department of Health, formally adopted a decentralised clinical training (DCT) strategy for undergraduate health sciences. Also known as Community-Based Training within a Primary Healthcare Model, DCT hopes to, in part, answer to the health systems development needs of the second most populous province in the country. It aims to address the notion of ‘fit-for-purpose’ graduates, where anecdotal evidence is that new health graduates employed within the public health sector face significant challenges relating to health care delivery within under-resourced environments.

In the DCT model, clinical training for senior health science students takes place away from academic centres where the majority of clinical training has historically taken place for most disciplines. UKZN is the first of the four institutions in the country offering optometry training to formerly adopt DCT as an adaptive clinical training strategy, in response to the aforementioned global and national developments. The initiative is also aligned with the need to strengthen eye health service delivery in the province, with the burden of non-communicable diseases in South Africa fostering a shift towards a developmental, PHC approach (Hofman, 2014). DCT also inherently acknowledges the link between poverty and ill-health, with the response supporting government’s efforts towards more comprehensive, integrated, accessible health services from primary to specialist care levels (National Department of Health South Africa, 2015). The intervention described in this paper forms part of a larger research project to explore the development of a socially responsive approach to training optometrists in South Africa within a community-based, PHC model.

## DCT in optometry: the UKZN experience

DCT in optometry was piloted 2016, with final (fourth) year optometry students placed at public health facilities under the supervision of resident clinicians across the KwaZulu Natal province. In 2016 and 2017 rotations, students were rostered in two to three-week placement blocks, respectively. Students were housed in or near the communities they served, which forms part of the strategy of giving students an appreciation of the community context within which they work, as well as determinants of health within the South African context. Placements were primarily at the district or regional hospitals, where treatment of patients was the primary goal. Rotations also included a weekly community component with students conducting outreach and screening services in un- or under-served areas (schools, outlying clinics and in some cases very rural, temporary health posts). This community engagement gave students exposure to health education and promotion activities which has previously been weak in optometry’s undergraduate training programme at UKZN. Patients who required further assessment and treatment would then be referred to the health facility for more comprehensive care.

Student experiences varied greatly, where at some facilities the resident optometrist worked within a comprehensive eye health team consisting of an ophthalmologist and/or medical officer, ophthalmic nurses and other support staff with far more than the basic required equipment for primary optometric care, and in other cases where the optometrist was the only eye health worker within a facility having access to minimal clinical equipment. This contrast was mostly viewed in a positive light, with students reporting having to apply a case-based approach to patient care, being creative about adapting procedures to these contexts, as well as having an appreciation for the inequality facing eye health service provision across the province (UKZN Optometry students, 2016).

As with any new initiative, there were initially mixed feelings in relation to DCT and its potential impact on service delivery from clinical staff. Introducing the student training component to the eye clinic’s operations created some unease in the pilot year with the lack of resources, most notably space and sufficient equipment, being a concern (Gangat *et al*., 2016). One pilot site, however, demonstrated that the inclusion of students within the service chain significantly increased the number of patients seen during the calendar year, almost doubling that of historical numbers. However, the students did require an initial adjustment and orientation period which initially slowed the usual flow of clinic operations; but improved once students became accustomed to the public sector’s system of work. Other clinicians reported that student involvement enabled them to elevate their level of contribution in case- and clinical service management, where students focussed on more basic procedures and clinicians could then engage in analysis of cases with students or clinical service development.

## A developmental eye health strategy?

The implementation of DCT in optometry is still in its infancy, with several key components around a revised PHC-centred curriculum and adaptive clinical teaching approaches yet to be defined. However, early indications are that the strategy has the potential to improve eye health services within the public sector in KwaZulu Natal. However, several considerations will need to be made for the initiative to be scaled up to its full potential.

### Improved access to eye health services

The inclusion of optometry student clinicians in the eye health service chain within KwaZulu Natal appears to be contributing towards addressing service backlogs on public-sector-dependent communities. However, as DCT in optometry becomes more fully integrated into the academic training of undergraduate students, securing sufficient numbers of sites may be a short-term limitation as budgetary constraints may impede the ability of the Department of Health to employ optometrists or equip service centres in all identified areas of need.

### Sensitisation of need

The optometry experience has shown that through the process of negotiating the placement of students at various facilities across the province, health officials are being sensitised to the needs around eye health service provision. Advocacy for optometry service development remains a central part of the DCT programme and its future success. Furthermore, increased awareness amongst health officials of the benefits of improved eye health service delivery through student involvement will likely serve as an advocacy tool for development of new service sites and/or increased employment of optometrists within the public sector.

Reflective feedback from optometry students is that the experience has sensitised them to the unmet need for eye health services within the public sector. This is fuelling their role as advocates for the ongoing development of eye health services nationally (UKZN Optometry students, 2016). The immense need encountered by students visiting community outreach sites also served to entrench a willingness to contribute to addressing the health needs of the country in general, and will likely attract more qualified candidates to the public sector, with many expressing the desire to follow this path.

### Development of new service sites

In the current model, students are placed at largely established centres of care which meet minimum academic and regulatory requirements for undergraduate optometry training (equipment, human resources), as most of the more rural hospitals either do not have optometrists or are currently insufficiently equipped to address training needs. However, the intention is that students will increasingly be placed in more outlying areas to address service gaps. With this in mind, engagement has already taken place with strategically identified sites who did not meet the minimum training needs at the time of the pilot and indications are that these sites are being capacitated to facilitate student placement in the near future.

### Clinical competency development

Optometry students reported that the DCT experience, which afforded them the opportunity to practice within a context of multiple challenges, including managing larger volumes of patients and seeing a wider scope of clinical conditions than they are exposed to at the academic clinic, supported practice-readiness. They also reflected on the opportunity as one that helped shape their professional identity. An audit of clinical exposure numbers in 2016 and 2017 confirms that the DCT programme has contributed towards students meeting or exceeding the minimum patient numbers and clinical hours required for professional registration much sooner than in previous years. The clinical training component, therefore, appears to be strengthened through DCT, ultimately making for more practice-ready clinicians. While access to specialised equipment presented a challenge in some instances, the overall experience reportedly made students more well-rounded clinicians.

### Context-appropriate training

DCT improved optometry students’ understanding of health care provision from a holistic, developmental perspective. It exposed them to the realities and socio-economic challenges facing disadvantaged communities, inequalities within health service delivery as well as health systems constraints within South Africa. These are all important contextual underpinnings in order to deliver a socially responsive and socially accountable health care service (Lee and Sadana, 2011). For some, this experience will likely develop leaders who will advocate for the health system and service delivery improvements in future. Students confirmed that pre-practice exposure to the public health sector through DCT fosters context-appropriate training. This initiative, therefore, appears to support broader competency development as defined by the Health Professions Council of South Africa (2014).

### Peer engagement and knowledge sharing

Joint supervision and training of undergraduate students’ by resident clinical staff at public health facilities and academic staff at UKZN presents an opportunity for mutual professional knowledge development, and skills transfer between public sector clinicians and academic staff. This is a win-win in a programme which merges two very different, but inter-related practice contexts. Furthermore, the potential for interprofessional care, while largely unexplored as yet unexplored, exists and needs to be developed and nurtured within the DCT programme.

## Conclusion

DCT in optometry shows promise in terms of enhancing the quality of the student’s clinical training experience, facilitating context-appropriate training and developing fit-for-purpose graduates. It further appears to hold a benefit for eye health service development and improved access to eye care within the public health sector in KwaZulu Natal. By taking promotive and preventative interventions into the unserved communities, DCT supports the notion of ‘taking health care to the people’ through a community-based PHC-centred training approach. Advocacy for DCT also invariably promotes sensitisation around discipline-specific service development needs on the part of both public health officials and future clinicians.

Historical and current unmet needs for eye care within the public health sector can potentially be addressed through this adaptive model of training, which simultaneously addresses capacity challenges at higher education institutions. However, while the initiative appears to have several potential benefits, institutions must be cognisant that the service delivery agenda should never overshadow the learning experience of students. The model also appears to support the development of graduate competencies for managing health care delivery in line with the Health Professions Council of South Africa’s ‘Core Competencies for Undergraduate Students health sciences’ (Health Professions Council of South Africa, 2014). However, this would require the development of more specific learning outcomes and assessment activities in this regard.

Throughout South Africa and Africa, there is a need for optimising eye health service delivery and increasing the number of eye health professionals serving rural communities. It is hoped that through DCT, optometry students will be better equipped to address public health system-related challenges facing eye health services in South Africa. Together with stronger clinical skills and more appropriate competencies developed through DCT, optometrists can in future serve as the advocates, leaders, managers and collaborators who will contribute towards developing the local eye health service. Through this model, targeted health education and promotion at the community level will also positively impact the burden of vision impairment, ultimately improving eye health outcomes for KwaZulu Natal in the long term.

If DCT in optometry lives up to its noble intentions and indicative potential, eye health services in KwaZulu Natal will be strengthened and likely have greater reach within rural communities. However, a socially responsive approach to eye health planning and service delivery is necessary for the strategy to be maximally successful, which is yet to be developed.
